# Dengue Fever: Causes, Complications, and Vaccine Strategies

**DOI:** 10.1155/2016/6803098

**Published:** 2016-07-20

**Authors:** Niyati Khetarpal, Ira Khanna

**Affiliations:** ^1^International Centre for Genetic Engineering and Biotechnology, Aruna Asaf Ali Marg, New Delhi 110067, India; ^2^Department of Biochemistry, University of Delhi, Institute of Home Economics, Hauz Khas, New Delhi 110016, India

## Abstract

Dengue is a highly endemic infectious disease of the tropical countries and is rapidly becoming a global burden. It is caused by any of the 4 serotypes of dengue virus and is transmitted within humans through female* Aedes* mosquitoes. Dengue disease varies from mild fever to severe conditions of dengue hemorrhagic fever and shock syndrome. Globalization, increased air travel, and unplanned urbanization have led to increase in the rate of infection and helped dengue to expand its geographic and demographic distribution. Dengue vaccine development has been a challenging task due to the existence of four antigenically distinct dengue virus serotypes, each capable of eliciting cross-reactive and disease-enhancing antibody response against the remaining three serotypes. Recently, Sanofi Pasteur's chimeric live-attenuated dengue vaccine candidate has been approved in Mexico, Brazil, and Philippines for usage in adults between 9 and 45 years of age. The impact of its limited application to the public health system needs to be evaluated. Simultaneously, the restricted application of this vaccine candidate warrants continued efforts in developing a dengue vaccine candidate which is additionally efficacious for infants and naïve individuals. In this context, alternative strategies of developing a designed vaccine candidate which does not allow production of enhancing antibodies should be explored, as it may expand the umbrella of efficacy to include infants and naïve individuals.

## 1. Introduction to Dengue

*(1) Overview*. Dengue is an infectious disease caused by any of the four dengue virus serotypes: DENVs 1–4. It is a mosquito-borne disease and is primarily transmitted to humans by the female* Aedes* mosquito. The disease is mainly concentrated in tropical and subtropical regions, putting nearly a third of the human population, worldwide, at risk of infection [1]. Infection with DENV results in varying degrees of pathological conditions, ranging from mild asymptomatic dengue fever (DF) to severe dengue hemorrhagic fever (DHF) and dengue shock syndrome (DSS) which may turn fatal [2]. A dramatic worldwide expansion of the DENV has occurred due to rapid urbanization, increase in international travel, lack of effective mosquito control measures, and globalization [3]. Though there is no approved drug, an update by Sanofi Pasteur reveals licensure of its vaccine in Mexico, Brazil, Philippines, and El Salvador [4].

*(2) Epidemiology*. Dengue has become one of the most widespread reemerging mosquito-borne diseases globally. Incidence of dengue has increased 30-fold in last five decades [5]. Currently, dengue is endemic to 128 countries, mostly developing nations, posing a risk to approximately 3.97 billion people annually. A recent dengue distribution model has estimated 390 million dengue infections annually, out of which 96 million cases occurred apparently [6, 7]. The Indian subcontinent is the epicenter of dengue [8] with cases being heavily underestimated [9]. Thus, there is an urgent need of improvement in serosurveillance to enable the authorities to prepare adequately for an outbreak.

*(3) Vector*. Dengue viruses are transmitted in humans by female* Aedes* (*Ae*.) mosquitoes of the subgenus* Stegomyia*.* Ae. aegypti* has been the most important epidemic vector in the tropical and subtropical regions. Other species such as* Ae. albopictus*,* Ae. polynesiensis*, member of* Ae. scutellaris* complex, and* Ae. niveus* have been found to play a role as secondary vectors [8]. However,* Ae. niveus* is considered only as a sylvatic vector. The life cycle of* Aedes* mosquito depending upon the extent of feeding lasts for 8–10 days at room temperature. It consists of two phases: aquatic (larvae, pupae) and terrestrial (eggs, adults) phase. Presently,* Ae. albopictus* has become an increasingly important vector as it can easily adapt to new environments, including temperate regions. Its spread to* Ae. Aegypti* free countries has created opportunities for dengue viruses to enter new locations and cause disease [10]. However, it is still a minor contributor to human dengue infections.

## 2. Dengue Virus

### 2.1. Genomic Structure

**Figure 1:**
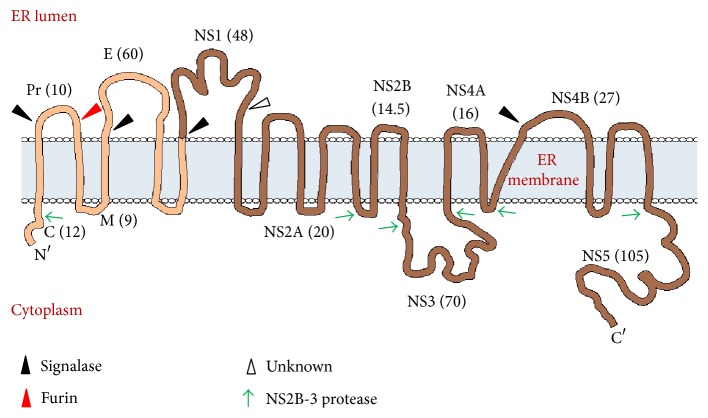
Genome organization and membrane topology of dengue virus. The viral RNA is translated as a single polyprotein consisting of structural (light brown-C, prM, and E) and nonstructural (dark brown-NS1, 2A, 2B, 3, 4A, 4B, and 5) protein components. Symbols C, prM, E, NS, and PM denote capsid protein, precursor membrane protein, envelope protein, nonstructural proteins, and plasma membrane, respectively. This single polyprotein then gets processed by viral (green arrow) and host (black arrow) proteases. The structural proteins (prM and E) remain anchored on the luminal side of the ER membrane. The C protein is anchored on the cytoplasmic side of ER membrane. prM is later cleaved by furin (red arrow) in the TGN into the pr peptide and M protein. The NS proteins are mainly processed by NS2B-NS3 (viral protease) in the cytoplasm. NS2A/2B and NS4A/4B are transmembrane proteins and thus stay anchored in the ER. The approximate molecular weight (in kDa) of each protein has been indicated in braces.

The viral genome consists of a positive sense RNA of ~11 kb. This RNA is translated into a single polyprotein which encodes for three structural proteins, namely, capsid (C), premembrane (prM), envelope (E), and 7 nonstructural proteins (NS1, NS2A, NS2B, NS3, NS4A, NS4B, and NS5) (Figure 1).

It consists of a single open reading frame and two noncoding regions (NCRs) at the 5′ and 3′ ends. It is expressed as a single polyprotein precursor, which is co/posttranslationally cleaved by viral and host proteases (Figure 1). The 5′ and 3′ NCRs contain secondary structures and conserved sequences, which are involved in regulation of viral replication. The 5′UTR (~100 nucleotides) has a type I methylated cap structure (m7G5′ppp5′A) but the 3′UTR (~450 nucleotides) lacks a terminal polyadenylate tail. Protein synthesis occurs in the cytoplasm on the Rough Endoplasmic Reticulum (RER), and the structural proteins get anchored to the ER on the luminal side, where assembly and maturation of virion occur (Figure 1) [2, 11]. Major functions of all the proteins are summarized in Table 1 [11, 12].

### 2.2. Structure of Virion and Envelope Protein

**Figure 2:**
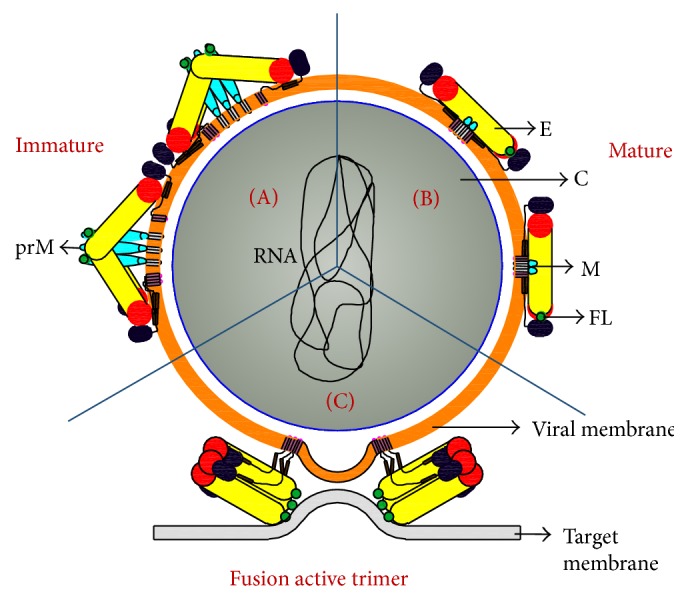
Organization of E protein on dengue virus surface during its life cycle. The E protein is colored as follows: EDI (red), EDII (yellow), EDIII (blue), and the FL (green). prM and M protein are colored as cyan. (a) Immature virus contains 60 trimeric spikes of E and prM heterodimer. (b) Mature virus contains 90 homodimers of E protein. (c) These homodimers then further undergo reorganization to form fusion active E homotrimers in which fusion loop is exposed. M protein is not shown in the fusion trimer for simplicity. E, C, M, FL, and prM denote envelope, capsid, membrane, fusion loop, and precursor membrane, respectively.

A three-dimensional image reconstruction of mature dengue virus shows that it is ~50 nm in diameter and consists of an outer protein shell (E and M), a lipid bilayer, and a less characterized nucleocapsid core (C and RNA genome). Dengue virus exhibits different surface structures during its maturation and infection and these conformational changes are attributed to the inherent flexibility of the envelope protein. E protein is made up of three domains, namely, EDI (red), EDII (yellow), and EDIII (blue), and transitions between its oligomeric states are supported by the hinge motion that occurs between EDI-EDII and EDI-EDIII. The immature virus particle has a spiky appearance with 60 trimeric surface spikes each consisting of three prM-E heterodimers (Figure 2). The pr peptides are cleaved in the TGN by furin, which leads to rearrangement of the E proteins into 90 homodimers of E that lie flat against the viral surface giving a smooth appearance, a characteristic of mature virus. In an E dimer, the E monomers are arranged face to face with their long directions being antiparallel to each other. M remains anchored to the lipid bilayer below the E protein shell (Figure 2).

When mature virus infects a new cell through receptor-mediated endocytosis, the E protein molecules shift to a trimeric conformation in the acidic compartment of an endosome, protrude from the virus surface causing membrane fusion, and facilitate viral RNA release into the cytoplasm (Figure 2) [2, 13, 14].

The E protein is the major exposed antigen of the dengue virion, antibodies against which provide immunity during natural infection. The E proteins of the four DENV serotypes have 60–70% amino acid similarity and are glycosylated at Asn-67 (unique to dengue) and Asn-153. These residues have been found to play important roles in the receptor attachment and viral entry into the cell. The E protein consists of a transmembrane region and an ectodomain which is divided into three structural/functional domains [15]:

- EDI (envelope domain I), central region, contains 8-stranded *β*-barrel and organizes the structure.
- EDII (envelope domain II) is a dimerization domain and contains 12 *β* strands, 2 *α* helices, and a highly conserved fusion loop.
- EDIII (envelope domain III) contains immunoglobulin like domain with 10 *β* strands and is involved in receptor binding.

**Figure 3:**
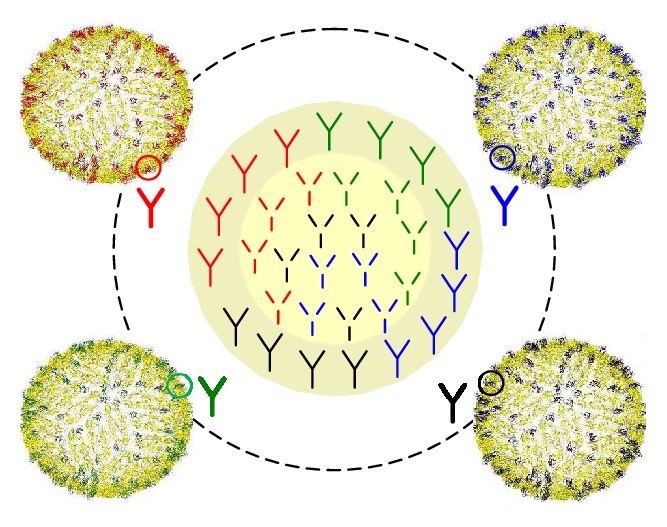
DENV-1, DENV-2, DENV-3, and DENV-4 with EDIII (circled) in red, green, blue, and black, respectively. EDIIIs elicit strongly neutralizing serotype-specific antibodies (antibodies in bold). Owing to homology especially in domains (EDI/II and prM) in yellow, cross-reactive weakly neutralizing antibodies (unbold antibodies) and nonneutralizing enhancing antibodies (dashed antibodies) are elicited in bulk.

Apart from the primary difference in their structure, the three domains of the ectodomain differ in their immunogenicity as illustrated schematically in Figure 3. The EDIII of each of the four serotypes (circled and colored in red, green, blue, and black for DENV-1, DENV-2, DENV-3, and DENV-4, resp.) elicits strongly neutralizing antibodies, which are largely serotype-specific [16]. Strongly neutralizing serotype-specific antibodies have been largely found to be elicited against the EDI/II hinge region [17], complex quaternary epitopes displayed on the E protein dimer and the whole virion [18, 19]. It has been reported that it is serotype-specific neutralizing antibodies, and not cross-reactive neutralizing antibodies, that confer protection against infection [20]. It has been reported that bulk of the immune response is elicited against the cross-reactive domains of EDI, EDII, and prM (yellow domain in the virus image) [16, 21]. Anti-EDI/II antibodies (Figure 3, unbold antibodies) are largely heterotypic weakly/nonneutralizing, while prM antibodies (Figure 3, dashed antibodies) are largely nonneutralizing and cross-reactive. It is believed that the virus utilizes such weak/nonneutralizing cross-reactive antibodies in gaining access into the host cell via Fc receptor as an alternative pathway during a secondary infection with a heterologous serotype, leading to enhancement of infection [16, 20, 21]. This phenomenon is known as antibody-dependent enhancement (ADE).

Thus, it can be inferred that strongly neutralizing antibodies prevent ADE caused by a larger population of weakly/nonneutralizing enhancing antibodies. A vaccine candidate capable of a larger population of strongly neutralizing antibodies could probably be an ideal vaccine showcasing strong protection without ADE. This can probably be achieved by designing the vaccine candidate and not by default strategy.

### 2.3. Cellular Targets and Receptor Interaction

**Figure 4:**
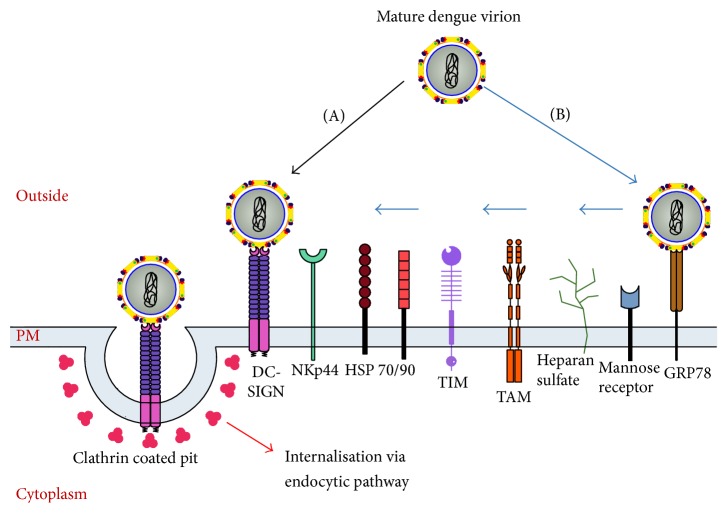
Schematic representation of the dengue virus entry process. The dengue virus makes use of membrane receptors and attachment factors on the cell plasma membrane (PM) to find its way to the cytoplasm. The mature virion either gets attached directly to a cellular membrane receptor (a) or uses several attachment factors (b) to finally trigger the endocytic, clathrin dependent pathway. The endocytic vesicle becomes a late endosome, where acidification triggers conformational changes on the E protein dimers to become fusogenic trimers. Finally, pores are formed and the genome of the virus is released into the cytoplasm.

The current model of flavivirus cell entry suggests the use of two functionally different sets of molecules: attachment factors that help the virus to concentrate on the cell surface and primary receptor(s), which help directing the virion to the endocytic pathway (Figure 4) [22].

During natural dengue infection in humans, the mosquito delivers virus in skin epithelium where it infects and replicates in the cells of mononuclear lineage like monocytes, dendritic cells, macrophages, and Langerhans cells [23, 24]. These infected cells carry the virus to lymph nodes, where it replicates, resulting in viremia, which is followed by systemic infection of liver, lungs, and spleen. However, in mosquitoes, the primary target of DENV infection is the epithelium of the midgut, where it first replicates and then [25, 26] spreads to and replicates in salivary glands, from where the infection is transmitted through saliva to the next vertebrate host during the blood meal.

## 3. Dengue Disease

### 3.1. Classification

The spectrum of clinical illness may range from asymptomatic disease to a broad range of syndromes with severe clinical manifestations. Symptomatic infection may range from mild debilitating DF to life threatening DHF and DSS due to plasma leakage in DHF patients. These three conditions likely represent progressively severe stages of a continuous dengue disease spectrum [27]. They are based on traditional WHO classification case definitions and continue to be recognized in many regions of the world despite the introduction of a new classification system. The new classification based on a single parameter [5] allows better case capture [28] but is not compatible with restricted health care facilities in endemic regions, especially during outbreaks [29].

### 3.2. DF

DF is a self-limiting fever, lasting usually for 5–7 days. It is sometimes debilitating during the acute illness stage. The clinical features of DF vary according to the age of the patient. The infants and young children may have undifferentiated febrile sickness with maculopapular rash. The older children and adults may have mild febrile syndrome or severe disease with high fever (usually biphasic), severe headache, retroorbital pain, myalgia, arthralgia, nausea, vomiting, and petechiae. Leukopenia and thrombocytopenia are usually observed in all ages. In some cases, DF may accompany bleeding complication such as gingival bleeding, epistaxis, gastrointestinal bleeding, haematuria, and menorrhagia (in case of women) [27].

### 3.3. DHF/DSS

DHF is characterized by symptoms of DF along with thrombocytopenia, hemorrhagic manifestations, and plasma leakage. A positive tourniquet test may be suggestive of DHF; however, this is being debated now due to its low sensitivity/specificity. Plasma leakage determines disease severity in DHF. It is also the most important difference between DHF and DF. Depending on disease severity and clinical manifestations, DHF is divided into four grades I to IV, with grade IV being the most severe. Several patients also have fine petechiae scattered on the extremities, axillae, face, and soft palate, usually seen in the febrile period. The critical phase is usually reached at the end of febrile illness, marked by rapid decrease in temperature and often accompanied by circulatory disturbances including plasma leakage, hemoconcentration, and thrombocytopenia [27].

In severe cases, with critical plasma loss, DSS ensues and may be life threatening if not treated properly. DSS is characterized by a rapid, weak pulse with narrowing pulse pressure (<20 mm of Hg), cold clammy skin, and restlessness. The patient may die within 12–24 h of going into shock or recover rapidly with volume replacement therapy.

### 3.4. Primary and Secondary Dengue Infection

The first exposure of an individual to any of the four dengue virus serotypes is known as primary dengue infection. It may/may not result in symptomatic infection. In primary infection, high titers of immunoglobulin M (IgM) and immunoglobulin G (IgG) antibodies appear in 3–5 and 6–10 days, respectively, after the onset of infection. The presence of IgM is transient, disappearing in 2-3 months after the onset of illness, whereas IgG persists for life [30]. Hence, primary infection with a particular serotype provides life-long immunity against that serotype. But it does not provide continued cross-protective immunity against the remaining serotypes.

A secondary infection, with a previously unencountered DENV serotype, usually results in classical DF. However, 2-3% of secondary infection cases develop into DHF, which may progress to DSS and death. During a second infection with a different serotype, the presence of low amounts of heterotypic antibodies (which form complexes with DENVs) promotes the access of the virus to monocytes, via Fc receptors, leading to an increase in viral load and severity of the disease. This phenomenon is known as ADE. The major players of this phenomenon are cross-reactive antibodies elicited against the fusion loop and prM, which are found to be weakly neutralizing leading to enhancement of infection at low concentrations [16, 21]. Although ADE has been found to result in disease severity, all the severe cases are not associated with secondary infection nor do all the cases of secondary infection progress to DHF/DSS [2]. In addition to humoral immunity, cross-reactive memory T cells could also play a role in either providing protective immunity or causing immunopathology [31].

### 3.5. Diagnosis and Clinical Management

Dengue infection is usually confirmed by identification of viral genomic RNA, antigens, or the antibodies it elicits. Antigen detection tests based on NS1 detection have been designed to detect the dengue viral NS1 protein which gets released from the dengue infected cells and appears early in the bloodstream. A 3-in-1 test for simultaneous detection of NS1, IgM, and IgG is now available. ELISA-based serological tests are easy to perform and are cost-effective for dengue detection.

Up to date, there is no antiviral drug available for dengue. Treatment is usually based on symptoms and is performed through medical support. For uncomplicated cases of dengue fever, the treatment prescribed is bed rest, oral rehydration, and paracetamol as an antipyretic and analgesic. Patient's health is monitored through various blood tests from fever day 3 onwards till the condition improves. Clinical signs that signal progression to serious disease include cold limb extremities, low pulse, low urine output, signs of mucosal bleeding, and abdominal pain. DHF is indicated by a rising hematocrit (≥20%) and a falling platelet count (>100,000/mm^3^). If any of these signs are detected, immediate hospitalization is necessary. Treatment for DHF patients is based on intravenous fluid therapy to maintain effective circulation during plasma leakage plus careful clinical monitoring of hematocrit, platelet count, pulse rate and blood pressure, temperature, urine output, fluid administered, and other signs of shock. Patients usually recover within 12–48 h of fluid therapy. Treatment for DSS patients mainly consists of immediate fluid therapy with colloids and extensive monitoring of any complications. In worse case such as internal hemorrhage, whole blood transfusion may be carried out [27].

## 4. Dengue Vaccine Strategies

**Figure 5:**
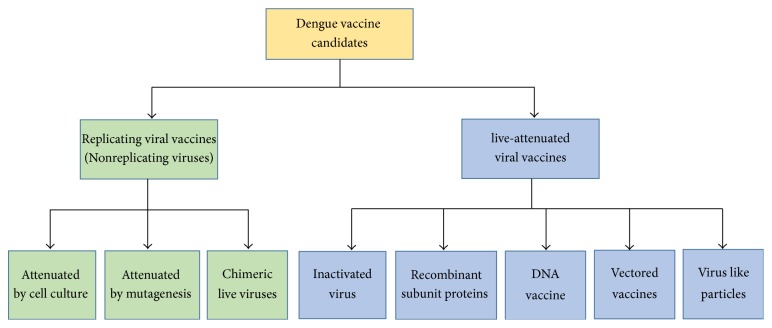
Classification of dengue vaccine candidates.

Despite the existing challenges for an ideal dengue vaccine, development of dengue vaccine candidates has progressed over the last decade and some of these have entered clinical trials in both endemic and nonendemic areas. A classification of the current approaches for dengue vaccine development is shown in Figure 5.

### 4.1. Replicating Viral Vaccines

These include live-attenuated viruses (LAV) that are created by reducing the virulence of a pathogen without compromising its viability. Current methods of producing live-attenuated viruses for dengue vaccines include attenuation by serial passage in cell lines and targeted mutagenesis and by constructing chimeric vaccine viruses:

- Advantages: robust, lasting, and broad immunity and lower production cost.
- Disadvantages: difficulty in attenuation, genetic instability, possibility of reversion, and interference in the case of multicomponent LAV vaccines.

#### 4.1.1. Cell Culture Passage Based LAV

Development of LAV by serial passage in cell lines was started at Mahidol University, Bangkok, Thailand. A tetravalent formulation was made by attenuating all four DENV serotypes, but the vaccine failed to elicit a balanced immune response despite modulating the viral concentrations [32, 33]. Increased frequency of adverse reactions like fever, rash, myalgia, and retroorbital pain, primarily related to the DENV-3 vaccine strain, was observed. Further development of these LAV strains was stalled [34].

Another LAV, based on passaging in cell culture, was developed by Walter Reed Army Institute of Research (WRAIR), Maryland, USA, and is being evaluated in clinical trials in collaboration with GlaxoSmithKline (GSK). All four DENV serotypes were attenuated by passaging in primary dog kidney (PDK) cells, and a tetravalent formulation (F17/Pre) was developed which was found to result in DENV-4 vaccine-induced viremia during phase II clinical trials [35, 36]. In a separate phase II randomized observer-blind, placebo-controlled trial in 86 healthy flavivirus naïve adults in USA, F17/Pre DENVs were rederived and passaged in fetus rhesus lung cells to obtain seed viruses of higher purity. Resultant formulations F17 and F19 containing equivalent amounts of vaccine components, except DENV-4 being 10-fold higher in F17, were evaluated. An acceptable safety and immunogenicity profile was observed after 2 doses of LAV with tetravalent antibody rates of 60% and 67% in participants receiving F17 and F19, respectively. It was also reported that although F19 was formulated to contain 10-fold less DENV-4, it was found to be only fourfold less at the time of vaccine release. The neutralization titers against DENV-4 were found to be comparable at 70 and 46 for F17 and F19, respectively. Notably, the incidence of DENV-4 vaccine-induced viremia reduced (with only one case in F17 group) probably due to rederivation and passage which attenuated the DENV-4 strain further [37]. In a similar phase II trial in healthy children and adults in Puerto Rico, F17 and F19 were evaluated again. The DENV-4* in vitro* potency in F19 was found to be 50-fold less, instead of being 10-fold according to formulation design. Thus, there have been issues related to storage stability of DENV-4 strain [38].

#### 4.1.2. Targeted Mutagenesis Based Live-Attenuated Vaccine

This strategy was first successfully explored by the Laboratory of Infectious Disease at the National Institute of Allergy and Infectious Disease (NIAID), National Institutes of Health (NIH), Maryland, USA. NIH has established nonexclusive license with manufacturers in Brazil (Instituto Butantan), Vietnam (Vabiotech), and India (Serum Institute of India and Panacea Biotech) for its development. This vaccine candidate is a mixture of four DENV strains attenuated by site directed mutagenesis to delete 30 nucleotides in the 3′UTR. DENV-1 and DENV-4 attenuated strains were designated as DEN1Δ30 and DEN4Δ30, respectively [39]. DENV-2 and DENV-3 attenuated strains were made by using DEN4Δ30 as a backbone and replacing their structural prM and E genes with those of the corresponding serotype. Notably, chimerization resulted in overattenuation of rDEN2/4Δ30 and rDEN3/4Δ30 strains. The DENV-3 component was modified variably and rDEN3Δ30/31 strain was selected where additional 31 nucleotides were deleted from rDEN3Δ30. Infectivity of DENV-2 component has been improved in tetravalent formulation TV005 by using DENV-2 attenuated rDEN2/4Δ30 strain at a 10-fold higher dose (10^4^ pfu) than other components (10^3^ pfu); tetravalent formulation TV003 contains 10^3^ pfu of each of the four components. Importantly, a single dose of TV005 has been found to be efficacious in providing sterilizing immunity. Additionally, TV003/TV005 are being evaluated in a human challenge model to enable a more stringent assessment of its protective efficacy. TV003 has been found to protect vaccinees against challenge with DENV-2 attenuated rDEN2Δ30 strain [40, 41]. Similar evaluation of protective efficacy is ongoing for TV005 and DENV-3 human challenge experiments are being planned [40]. Phase III of this vaccine candidate has begun in Brazil [42].

#### 4.1.3. Chimeric Dengue Vaccine

Chimeric dengue vaccines have been designed using two approaches: (i) with another attenuated flavivirus and (ii) with an attenuated DENV strain (intertypic chimera). The vaccine where chimera of DENV has been made with another flavivirus is the chimeric yellow fever-dengue (CYD) vaccine, which is being developed by Sanofi Pasteur and licensed under the brand name “Dengvaxia” [4]. In this vaccine, prM and E genes of the attenuated yellow fever LAV strain 17D have been replaced with the corresponding genes from DENV [43]. The rationale behind this design was the fact that humoral response against the structural proteins of dengue was responsible for protective immunity during natural infection and thus these chimeras would generate a protective immune response in vaccinees. A tetravalent mixture of the four chimeric viruses has undergone extensive clinical evaluation and has recently been approved in Mexico, Brazil, El Salvador, and Philippines [4, 44]. This vaccine will be discussed in detail in the later sections.

An example of intertypic chimera is DENVax developed by Inviragen Inc., Fort Collins, CO, USA. DENV-2 strain attenuated by 53 passages in PDK cells (made at Mahidol University) has been used as a backbone for generating chimera. prM and E gene of this strain were replaced by corresponding genes from DENV-1, DENV-3, and DENV-4. As the mutations in the attenuated strain were in the nonstructural proteins, this strain was used as such for DENV-2 component in the tetravalent formulation. These chimeric viruses showed a temperature-sensitive phenotype, reduced replication in mosquito cell lines, high degree of genetic stability, and lack of neurovirulence in suckling mice [45]. Three tetravalent formulations with variable dose of each component were evaluated in nonhuman primates. It was observed that DENV-2 was the dominating component and its replicative potential reduced by increasing the DENV-3 and DENV-4 component. This variation in DENV-2 induced viremia due to the variation in the dose of DENV-3 and DENV-4 components indicated viral interference. Moreover, the neutralizing antibody titers were found to be significantly low against DENV-4 and despite this, macaques were found to be protected against DENV-4 challenge [46]. A phase I clinical trial of low and high doses of the DENVax in healthy subjects in Columbia revealed that the candidate was safe and immunogenic. Notably, it corroborated the findings made in nonhuman primate study that neutralizing antibody titers elicited by DENVax are lowest against DENV-4 and highest against DENV-2 [47]. Further insights into its efficacy will be revealed through its phase II clinical trial evaluation. Meanwhile, phase III evaluation of this vaccine has now been initiated [48].

### 4.2. Nonreplicating Viral Vaccines

These vaccine candidates are not capable of replicating and thus offer the advantage of conferring immunity without the risk of infection. There are multiple strategies to develop this class of vaccines like DNA vaccines, subunit proteins, VLPs, and so forth:

- Advantages: reduced reactogenicity, better suitability for immune-compromised individuals, and balanced immune response in case of tetravalent formulation.
- Disadvantages: less broad, potent, and durable immune response, which may result in ADE, and requiring the use of adjuvants.

#### 4.2.1. Purified Inactivated Virus (PIV)

WRAIR, Maryland, USA, developed an inactivated monovalent dengue vaccine by formalin treatment. It was found to be safe and immunogenic in mice and* Rhesus macaques* [49, 50]. Although such vaccines would not show viral interference or revert to a pathogenic strain, their use as the sole immunization approach is limited because of conformational changes in virus by formalin treatment and lack of replication. However, this vaccine has been tested as the priming vaccine in a prime-boost immunization strategy, with a LAV as the booster vaccine, leading to complete protection in macaques [51]. Phase I trial evaluating the safety of 2.5 and 5 *μ*g of DENV-1 component administered on days 0 and 28 in flavivirus naïve population in the USA has been completed [52]. Two phase I trials evaluating tetravalent mixture of the four PIVs (TPIV; 1 *μ*g of each of the four PIVs) are being evaluated with alum and two proprietory adjuvants of GSK (AS01E1 and AS03B1) in healthy adults in the USA [53] and in Puerto Rico [54]. Healthy adults in the USA are also being recruited in another phase I study where TPIV/alum is being evaluated in prime-boot vaccination with WRAIR/GSK's tetravalent LAV [55].

#### 4.2.2. Recombinant Subunit Vaccine

Recombinant E proteins of dengue have been expressed in yeast and insect expression systems and have been analyzed for vaccine efficacy in mice and monkeys. All these studies have focused on the DENV E aminoterminal 80% of the molecule known as the ectodomain. Deletion of 20% of the E protein at the C-terminal, which is a transmembrane region, allows extracellular secretion and easy purification while retaining its antigenicity. The recombinant 80% E proteins, also known as r80E, of the four DENV serotypes are being manufactured by Hawaii Biotech Inc., HI, USA, and Merck and Co., NJ, USA. Monovalent DEN2 80E was evaluated with a panel of adjuvants in mice and saponin-based adjuvant ISCOMATRIX*™* was found to be the most immunogenic; immunogenicity with alum as adjuvant was poor. This was followed by evaluation of tetravalent formulation with ISCOMATRIX in macaques, where titers against DENV-4 were found to be the weakest [56]. To overcome the low immunogenicity of DEN4 80E, its dimeric form and double dose were explored in macaques, which led to comparable improvement in the neutralizing titers against DENV-4. Though the titers against DENV-4 improved, they were lower than the titers against DENV-1, DENV-2, and DENV-3 [57]. Based on these results, a tetravalent mixture of the four r80Es containing 10, 10, 10, and 20 *μ*g of DEN1, DEN2, DEN3, and DEN4 80E, respectively, was further evaluated in flavi-naïve and dengue-primed macaques where it was found to generate a more balanced immune response against the four serotypes in 0-, 1-, and 6-month immunization schedule as compared to 0, 1, and 2 months. Moreover, two doses (10 and 50 *μ*g) of DEN1 80E/alum administered in flavi-naïve adults on 0, 1, and 2 months were found to be safe. However, it elicited only modest DENV-1 neutralizing titers which waned almost completely 26 weeks after the final dose [58]. A phase I study examining safety and immunogenicity of tetravalent formulation with and without adjuvant (alum and ISCOMATRIX) in healthy adults has been completed [59].

Recombinant antigens based on DENV EDIII have been produced by different groups using* E. coli* and yeast expression hosts. Recombinant EDIII antigens, expressed either independently or fused to different carriers such as maltose-binding protein [60] and the Neisseria meningitides p64k protein, have been shown to generate anti-DENV immune responses in mice and nonhuman primates [61–63]. These vaccines candidates are in preclinical phase currently.

#### 4.2.3. Dengue DNA Vaccine

This vaccine consists of a plasmid vector containing the gene(s) encoding for an antigen, which on immunization is taken up by antigen presenting cells (APCs). Once the plasmid enters the cell, it codes for the antigen which finally gets associated with MHC class I molecules and gets displayed on the cell surface, inducing protective cytotoxic immune response. Naval Medical Research Center (NMRC), USA, has developed a DENV-1 DNA vaccine candidate (D1ME^100^) by cloning prM and E gene of DENV-1 serotype into plasmid vector, which was extensively evaluated in mice and macaques without adjuvant [64] before phase 1 trials in healthy adults. Although DENV-1 DNA vaccine was found to be well tolerated, the neutralizing antibody titers and the number of responders were found to be low [64, 65]. Thus, to enhance its immunogenicity, a lipid-based adjuvant Vaxfectin was explored. Tetravalent dengue DNA vaccine (TVDV) was evaluated for immunogenicity with and without Vaxfectin in macaques. It was observed that Vaxfectin resulted in higher and more stable (evaluated till 6 months after final boost) titers. The average neutralization titers with TVDV/Vaxfectin against DENV-1, DENV-2, DENV-3, and DENV-4 a month after final boost were approximately 200, 270, 170, and 70, respectively. Six months after the final boost, the titers against DENV-2 and DENV-3 reduced, while those against DENV-1 and DENV-4 increased marginally. In the group without Vaxfectin, titers against DENV-2 only were detectable 6 months after the final boost. Moreover, Vaxfectin allowed better protection from viremia against DENV-2 challenge [66]. After establishing nontoxicity of TVDV/Vaxfectin in New Zealand white rabbits [67], a phase I trial was initiated in 2011 in USA [68].

#### 4.2.4. Replication-Defective Virus Vectored Vaccines

In this approach, a virus is used as a vector to carry antigenic genes that are capable of eliciting neutralizing antibody response. Some examples of viral vectors are adenovirus vectors, Venezuelan equine encephalitis virus vector, and attenuated measles virus [69–71]. An example of virus vectored dengue vaccine is cAdVax. It consists of bivalent constructs expressing prM and E proteins from two dengue serotypes each (DENV-1 and DENV-3 together in one and DENV-2 and DENV-4 in another construct). Study in NHPs showed production of neutralizing antibodies to respective DENV serotypes [72, 73]. Therefore, a tetravalent formulation (cAdVax-DenTV) was prepared by mixing the bivalent constructs, which showed protection against all serotypes on DENV challenge in* Rhesus macaques* [69].

#### 4.2.5. Virus Like Particle (VLP) Vaccines

The prM and E proteins of DENVs coexpressed in heterologous hosts have been shown to coassemble into VLPs. Thus, a vaccine based on physical mixtures of four monovalent DENV VLPs can be developed to have a tetravalent formulation [74]. From the perspective of using VLPs for vaccine purpose, the yeast system may be more suitable, as it has the potential for higher yields and can glycosylate the antigens. Recent work indicates that yeast expressed-DENV E ectodomain forms VLPs in the absence of prM [75]. Another approach is based on displaying the DENV EDIII on VLPs formed by hepatitis B virus core antigens [27].

## 5. Dengvaxia

Dengue vaccine candidates which have reached clinical trials are given in Table 2. It is worthwhile to discuss the front-runner CYD vaccine developed by Sanofi Pasteur which has recently been approved as Dengvaxia in Mexico, Brazil, El Salvador, and Philippines [4]. Dengvaxia is a tetravalent dengue chimeric live-attenuated virus vaccine, based on licensed yellow fever vaccine 17D. It was constructed by replacement of structural genes of live-attenuated yellow fever virus vaccine 17D with structural genes from each DENV serotype [76].

### 5.1. Preclinical Data

The immunogenicity of various tetravalent formulations of the chimeric viruses was evaluated in macaques, which revealed immunodominance of serotype 4 chimeric virus; neutralizing antibody titers in the elicited responses were consistently lowest against DENV-2 [77, 78] and they failed to confer solid protective immunity to wild dengue challenge [79].

### 5.2. Phase I Trial

Monovalent serotype 2 chimera, evaluated in a phase I study in healthy adults 18–49 years old, was found to be safe and immunogenic [80]. Therefore, tetravalent formulation containing 5 log_10_⁡ cell culture infective dose 50 (CCID50) was tested in dengue naïve US adults aged 18–45 years. The vaccine was well tolerated with all the participants seroconverting to all four DENV serotypes after receiving three doses of the vaccine. However, low levels of viremia were observed primarily against DENV-4 [81]. Another phase I trial was conducted in dengue endemic area like Philippines. Here, the vaccine was evaluated on subjects of four age cohorts: 2–5, 6–11, 12–17, and 18–45 years. Vaccine was found to be safe and all the vaccinees exhibited high seroconversion rate (>88%) for all the four DENV serotypes [82]. Thus, the tetravalent vaccine was safe and immunogenic in both dengue endemic and nonendemic areas.

### 5.3. Phase II and Phase IIb Trials

A randomized, double-blind multicenter phase II trial was conducted in healthy US adults to test various tetravalent formulations for the CYD-TDV vaccine. Although all vaccine formulations were safe and immunogenic, the formulation containing 5 log_10_⁡ tissue culture infective dose 50 (TCID 50) of each serotype demonstrated the best immunogenicity. This formulation was used for further studies [83]. Another randomized, controlled phase IIb trial was conducted in 4–11-year-old school children at Ratchaburi Province, Thailand. The overall efficacy of CYD-TDV was found to be a low 30.2% (95% CI −13.4 to 56.6) after 3 doses. Moreover, the efficacy was highly variable between the various serotypes: 55.6% (95% CI −21.6 to 84.0) for DENV-1, 9.2% (95% CI −75.0 to 51.3) for DENV-2, 75.3% (95% CI −375.0 to 99.6) for DENV-3, and 100% (95% CI 24.8 to 100.0) for DENV-4 [84]. It should be noted that confidence intervals of all the efficacies except that against DENV-4 included zero, which raises concerns over the significance of these results.

The lack of efficacy against DENV-2 in this trial may be attributed to the following reasons:

- The genotype of DENV-2 circulating in Thailand had an antigenic mismatch with the vaccine virus strain due to mutations in E [85].
- PRNT assay used to determine the neutralizing antibody titers during the trials was carried out in Vero cells that lack the Fc*γ* receptors on the cell surface. As ADE can play an important role* in vivo* using these receptors, this assay may not truly predict vaccine efficacy [85].
- As the vaccine molecule contained many cross-reactive epitopes, therefore, it is possible that enhancement took over the neutralization potential of antibodies* in vivo* leading to poor efficacy, as was later observed during phase III trials too [86].

Another randomized, blinded, controlled phase II trial was conducted in 9–16-year-old subjects from Latin America. The seropositivity for at least two, three, or all four serotypes was 100%, 90.6%, and 93.4%, respectively, after 3 doses. Vaccinees, who were seropositive for flavivirus antibodies before immunization, had higher antibody titers upon immunization (as compared to seronegative subjects). The rates of virologically confirmed dengue cases for all four DENV serotypes were lower in the vaccine group compared to that in the control group. The contrast in results between this trial and the one conducted in Thailand was attributed to the difference in epidemiology and circulating virus strain differences between the two countries [87].

### 5.4. Phase III Trial

An observer-masked, randomized controlled, multicenter, phase III trial was done on healthy children aged 2–14 years in 5 countries of Asia-Pacific regions. They were randomly assigned (stratified by age and site) to receive three doses of CYD-TDV, or placebo, at 0, 6, and 12 months. Subjects were followed up until 25 months. The primary endpoint was achieved with 56.5% (95% CI 43.8–66.4) efficacy. Thus, the vaccine was found to be moderately efficacious. Though the overall efficacy improved, it remains low and statistically insignificant against DENV-2 at 35.0% (95% CI −9.2 to 61.0) [88]. A follow-up of the vaccinees in year 3 to score the relative risk of hospitalization for virologically confirmed dengue revealed alarming results for children between 2 and 5 years. The rate of hospitalization of vaccinees of this age group was more than seven times the control group. Overall, the relative risk of hospitalization for children <9 years was 1.58 as compared to the alarming 7.45 for 2–5-year-old children. Moreover, vaccine efficacy was also found to be lower in vaccinees <9 years of age. The overall vaccine efficacy was 67.8% (95% CI 57.5 to 75.6) and 44.6% (95% CI 31.6 to 55.0) for participants above and below 9 years of age, respectively. This difference in efficacy was more pronounced in dengue naïve participants, where overall efficacy was reported to be 61.6% (95% CI −21.1 to 88.1) and a poor 14.4% (95% CI −111 to 63.5) in participants above and below 9 years of age, respectively [89]. The outcome that CYD-TDV vaccine puts children <9 years of age at greater risk of hospitalization is a serious safety concern. It is believed that CYD-TDV sensitized the dengue naïve subjects of all the age groups (owing to its low efficacy) to enhanced dengue infection, increasing the risk of hospitalization. Although it was found to be efficacious in reducing the risk of hospitalization in seropositive recipients, it has been estimated that, for every two recipients prevented from hospitalization, one recipient was hospitalized due to vaccine-induced enhanced disease [86]. These concerns have put children <9 years of age and dengue naïve population outside the ambit of its application due to safety concerns and poor efficacy.

Another phase 3 efficacy trial of CYD-TDV was carried out in five dengue endemic Latin American countries. Healthy children between the ages of 9 and 16 years were randomly assigned in a 2 : 1 ratio to receive three doses of the vaccine or placebo at 0, 6, and 12 months under blinded conditions. The subjects were followed up for 25 months. Serotype-specific vaccine efficacy was found to be 50.3% (95% CI 29.1 to 65.2), 42.3% (95% CI 14.0 to 61.1), 74% (95% CI 61.9 to 82.4), and 77.7% (95% CI 60.2 to 88.0) for DENV-1, DENV-2, DENV-3, and DENV-4, respectively. A statistically significant efficacy against DENV-2 was a big boost. Though the overall efficacy of the vaccine in virologically confirmed dengue cases was 60.8% (95% CI 52.0 to 68), it was found to be low in dengue naïve population: 43.2% (95% CI −61.5 to 80.0) [90]. Since this study enrolled children 9–16 years old (9–11 and 12–16 years' cohorts), the relative risk of hospitalization was observed to be fairly low (0.53) in year 3 of the follow-up. But, consistent with the Asian-Pacific trials, the vaccine efficacy was found to be lower in dengue naïve vaccinees. The overall efficacy was 83.7% (95% CI 62.2 to 93.7) and a low 43.2% (95% CI −61.6 to 80.0) for dengue serotype positive and naïve vaccinees, respectively [89].

### 5.5. Licensed in Mexico, Brazil, and Philippines

Dengvaxia has received regulatory approvals in Mexico, Brazil, El Salvador, and Philippines for administration in adults aged 9–45 years [4] because of the increased risk of hospitalization observed in children <9 years old. Moreover, Dengvaxia was found to be poorly efficacious in naïve individuals which restricts its applicability to dengue endemic nations.

### 5.6. Challenges and Obstacles in Developing Dengue Vaccine

The lower efficacy of Dengvaxia against dengue naïve individuals has raised many issues on ADE. Most of the current vaccine candidates (e.g., LAV, inactivated virus, and chimeric viruses) carry all the cross-reactive epitopes, leading to generation of high quantity of cross-reactive antibodies (as compared to serotype-specific antibodies). Such an imbalanced response overwhelmed with poorly neutralizing cross-reactive antibodies can cause ADE, reducing the efficacy against the virus* in vivo*.

Recent study using AG129 mouse lethal model showed that inoculation with virus immune complexes (ICs) formed with high quantity of highly neutralizing cross-reactive Abs caused lethal infection even though peak viremia level was low. On the other hand, those formed with serotype-specific neutralizing antibodies (anti-domain III used in the study) did not cause any mortality at any concentration [20]. This indicates that serotype specificity of antibodies elicited can be crucial in deciding the efficacy of a vaccine candidate. However, recent data suggests that dengue vaccines are at a crossroad even with modest efficacy [89, 91]. Nevertheless, WHO recommends development of an alternative dengue vaccine candidate which is designed to elicit strongly neutralizing antibodies in absence of cross-reactive enhancing antibodies. Such a vaccine candidate would enable higher efficacy and applicability to a broader group of subjects including infants and naïve population.

## 6. Conclusion

The absolute need for an efficacious tetravalent DENV vaccine, lack of an adequate animal disease model, and immune correlates of diseases protection remain as some of the major obstacles in developing a successful dengue vaccine. Since the wild type mice do not replicate clinical signs of human dengue infection, genetically engineered mouse models have been developed with considerable success to mimic some aspects of human infection. The most successful system has been the use of mouse-adapted DENV-2 and AG129 mice that lack IFN-*αβγ* receptors. Due to suppression of IFN pathway, an important branch of host immune response is disabled, which allows DENV to replicate. AG129 mice on infection with mouse-adapted DENV-2 develop vascular leakage without neurological complications, thus mimicking human clinical signs of severe dengue. Moreover, this mouse model has been found to be useful in scoring ADE by passive transfer of anti-DENV antibodies and challenge with nonlethal dose of mouse-adapted DENV-2. The passively transferred antibodies are said to enhance the disease if the mice succumb to infection and die. Since mouse-adapted DENVs are not the naturally circulating strains, AG129 mice are being explored as a suitable dengue model with clinical isolates too [92]. With respect to evaluation of dengue vaccine candidates also, AG129 mouse model has been recommended by WHO. It should be noted that this model allows limited evaluation since it lacks both type I and type II IFN pathways. Hence, this limits production of high titer neutralizing antibodies which may further result in ADE [93]. Thus, extensive work is ongoing to further advance these mouse models to enable better extrapolation of mice data to humans.

Sanofi Pasteur dengue vaccine Dengvaxia has now been licensed in a few countries, but it recorded poor efficacy in dengue naïve individuals during phase III evaluation. This could be due to a number of reasons. It possesses yellow fever virus backbone and therefore lacks the critical dengue T cell epitopes of the nonstructural region, which have been reported to play a vital role in providing protection against dengue [94, 95]. Studies also implicate immunity to dengue NS1 to be essential in providing protection [96, 97], which it lacks. The observation that it led to enhancement of disease [86] indicated that it generates a lot of cross-reactive nonneutralizing/enhancing antibodies. Thus, not only the presence of DENV neutralizing antibodies but also DENV serotype-specific neutralizing antibodies may be the key to a successful dengue vaccine candidate. Predominant immune responses to a natural DENV infection are highly cross-reactive, in the presence of very limited serotype-specific neutralizing antibodies. This could be considered as immune evasion or disease enhancement strategy of DENVs. Immune responses elicited by most dengue vaccine approaches based on the whole virus may be similar to natural DENV infections and thus disease or immune enhancement strategies (predominant serotype cross-reactive neutralizing antibodies) of DENV may overcome the protective (minor serotype-specific neutralizing antibodies) efficacy of the whole virus based vaccine candidate. An effective dengue vaccine must be designed, which is capable of eliciting predominantly DENV serotype-specific neutralizing (protective) antibodies in the absence of serotype cross-reactive neutralizing (disease-enhancing) antibodies. The pipeline of dengue vaccines is growing and notwithstanding lower efficacy, a dengue vaccine may soon become available for human use.

## Figures and Tables

**Table 1:** Functions of DENV proteins.

Protein	Function
Structural	
Capsid (C)	Binds and stabilizes viral RNA
Premembrane/membrane (prM/M)	(i) Pr peptide functions as cap that protects the fusion peptide on E, thus preventing premature fusion (ii) M forms ion channel
Envelope (E)	(i) Recognition and binding to the host cell (ii) Involved in uncoating of virus by enabling fusion of viral and endosomal membranes

Nonstructural (NS)	
NS1	(i) Viral RNA replication
	(ii) Viral defense through inhibition of complement activation
NS2A	Viral replication and assembly
NS2B	NS3 protease cofactor
NS3	(i) Serine protease-cleaves viral polyprotein
	(ii) RNA helicase and RTPase/NTPase-viral RNA replication (iii) Induction of apoptosis in infected cells
NS4A	Induces membrane alterations and autophagy to enhance virus replication
NS4B	(i) Interacts with NS3-viral replication
	(ii) Blocks IFN-*α*/*β*-induced signal transduction and helps virus to escape host's innate immune response
NS5	(i) Methyl transferase domain
	(ii) RNA-dependent RNA polymerase

**Table 2:** Dengue vaccine candidates currently in different phases of clinical trials.

Type of vaccine	Developer	Phase
Chimeric yellow virus dengue vaccine (CYD)	Sanofi Pasteur	Licensed
Intertypic chimera-DENVax	CDC-Inviragen/Takeda	III
Targeted mutagenesis based LAV-TetraVax-DV	NIH	III
Cell culture based LAV	WRAIR-GSK	II
Purified inactivated vaccine-TDENV-PIV	WRAIR-GSK	I
Recombinant subunit vaccine-V180	Hawaii Biotech, Merck and Co.	I
DNA vaccine expressing prM and E protein	Naval Medical Research Centre, WRAIR	I
